# Developing Strategies for Sustainable Medical Equipment Maintenance in Under-Resourced Settings

**DOI:** 10.5334/aogh.2584

**Published:** 2020-04-13

**Authors:** Christina M. Webber, Gabriel Martínez-Gálvez, Manuela Lopera Higuita, Ephraim I. Ben-Abraham, Brent M. Berry, Maria A. Gonzalez Porras, Sara Aristizabal, Anders Asp, J. Luis Lujan, John W. Wilson

**Affiliations:** 1 Mayo Clinic Graduate School of Biomedical Sciences, Rochester, MN, US; 2 Department of Orthopedic Surgery, Mayo Clinic, Rochester, MN, US; 3 Department of Neurology, Mayo Clinic, Rochester, MN, US; 4 Department of Physiology and Biomedical Engineering, Mayo Clinic, Rochester, MN, US; 5 Department of Neurology, University of Minnesota, Minneapolis, MN, US; 6 Department of Biomedical Engineering, University of Texas at San Antonio, San Antonio, TX, US; 7 Well Living Lab, Inc., Rochester, MN, US; 8 Delos Living LLC, New York, NY, US; 9 Department of Neurologic Surgery, Mayo Clinic, Rochester, MN, US; 10 Department of Infectious Diseases, Mayo Clinic, Rochester, MN, US

## Abstract

Engineering technology plays a pivotal role in the delivery of health care in under-resourced countries by providing an infrastructure to improve patient outcomes. However, sustainability of these technologies is difficult in these settings oftentimes due to limited resources or training. The framework presented in this editorial focuses on establishing medical and laboratory equipment sustainability in developing countries and is comprised of four steps: 1) establishing reliable in-country relationships with stakeholders, 2) identifying needs for sustainable solutions locally, 3) exploring potential solutions and assessing their effort-to-impact ratios, and 4) working with strategic partners to implement solutions with clear performance metrics. By focusing on the sustainability of donated equipment instead of the equipment itself, this method presented distinguishes itself from other philanthropic endeavors in the field by seeking to establish preventive maintenance habits that can impact clinical outcomes of a community long term. Application of this methodology is reported in the Original Research Article “A Low-Cost Humidity Control System to Protect Microscopes in a Tropical Climate” by Asp et. al.

Engineering technology plays a pivotal role in the delivery of health care in under-resourced countries by providing an infrastructure to improve patient outcomes. Currently, the primary strategy for addressing the need for medical technology in underserved areas is to donate equipment and supplies [1]. This often lacks a strategy for sustainability. According to the World Health Organization (WHO), up to 70% of donated equipment in sub-Saharan Africa is not used effectively due to factors like lack of technical support and user training [1]. To illustrate, a functional piece of equipment can become incapacitated by something as simple as a missing bolt. If there is limited access to manufacturer-provided services, repair parts, and technical expertise, the initial short-term benefit is negated by subsequent dependence on outside organizations for continued donations [2]. Continued performance of any medical or diagnostic device requires a corresponding effort to build and maintain a proper infrastructure.

Sustainable strategies to optimize continued use of medical and diagnostic equipment are needed in under-resourced settings, including a focus on equipment repair and maintenance. This could include training local technicians to identify problems and repair equipment, as well as securing access to an appropriate supply chain of replacement parts. This editorial details the following steps for developing sustainable medical equipment repair and maintenance strategies: 1) establishing in-country relationships, 2) assessing the equipment and material needs locally, 3) exploring feasible solutions, and 4) implementing a plan and measuring potential impact.

## Establish in-country relationships

Existing relationships with non-profit organizations can be leveraged to establish introductory meetings and arrange visits with local healthcare facilities and laboratories. These relationships can also facilitate additional connections with government offices (e.g., Ministry of Health) and education sectors (e.g., local medical and technical universities with programs on medical device usage). An initial needs assessment visit may help to identify common or impactful equipment problems, develop relationships and communication with strategic collaborators. Developing in-country project collaborators throughout the process will help ensure proposed interventions will be both culturally and resource appropriate. Indeed, including local project collaborators as project co-sponsors may help build local autonomy and sustain project outcomes. Existing relationships can also be leveraged to enhance sustainability by establishing new avenues of supply chain for replacement parts and additional local or regional partners. Additionally, this collaboration across entities helps share the resource burden while also helping disseminate technical knowhow. There are many potential contacts that are not initially apparent, but are critical to the success of the project, including: transportation arrangements, import or custom management, and local medical equipment parts suppliers.

## Assess local needs

The initial needs assessment exploratory visit is also important to assess available medical and laboratory equipment, quality of personnel training, existing standard operating procedures, local infrastructure, and needs of the local communities. Understanding how both personnel and equipment are utilized on-site will help to develop a risk assessment of current protocols and continued performance. Previously established in-country relationships can help ensure a detailed appraisal of risks and community needs. This appraisal could be communicated to donating organizations to help inform future targeted donations.

Key topics to cover during the needs assessment include: equipment inventory lists (manufacturer, model, operational status, photographs, location, useful life expectancy, replacement cost, and availability of consumables), points of contact (name, job title, phone number, email), and existing protocols for equipment usage, maintenance, and repair. It is important to listen to the needs expressed by in-country colleagues as they use this equipment regularly. Information on personnel availability and the financial situation in-country will also prove to be necessary when assessing needs. A second, more focused needs assessment trip may be required to fully identify and characterize potential areas of impact.

## Prioritize focus and explore solutions

Equipment prioritization should be completed based on the team’s needs assessment with local input, accessibility to technical experts, complexity of equipment, international standards, effort-to-impact ratio, and relevance of the problem to in-country partners. Once target equipment is identified based on local needs assessment, possible solutions for maintenance and repair education should be evaluated. A coordinated, team-based approach with local partners ensures feasibility and capacity for appropriate solution implementation. It necessitates detailed research, outreach, communication, and patience. Multiple solutions may exist for each type of equipment and should be researched to ensure redundancy. Research into multiple solutions is more efficient in groups. It is important to establish clear deadlines and move forward in a timely manner in this phase. Information gathered can include: total cost of ownership, implementation challenges, preparation time, length of implementation, type of assessment metrics, and reproducibility. If a solution requires engaging local personnel, all members must have a clear understanding of personnel, time, and effort necessary for successful implementation. This information facilitates decisions about which plan to implement while accounting for in-country partners, chain of command, and cultural context, as well as the team’s capabilities and economic resources. It is, however, recommended to start with a straightforward solution even if it yields a small reward. This will help the team gain confidence, as well as establish credibility with partners.

## Implement plan and measure impact

Redundancy also plays an important role in the implementation phase. A main project objective should be to eliminate any dependency on a single individual or process that can halt implementation. For example, if the goal is to teach in-country partners to service and maintain equipment, then an ideal scenario would be to maximize the number trained during a single session, providing each person with the skills to teach others. A Train-the-Trainer model helps create a larger base of locally available experts, providing long-term project security. To enhance sustainability, be sure to include readily available supporting resources such as multimedia content, training videos, user manuals, maintenance tools, and/or replacement parts. Training can be a labor and resource intensive process, but it is critical for sustainable maintenance and increased longevity of medical devices. Ultimately, a sustainable solution should have a long-term impact and aim at independence. These tools will help work towards that.

Furthermore, metrics to assess efficacy of a strategy should be well defined prior to solution implementation, including assessment timepoints. Project metrics help gauge effectiveness and appropriateness of the intervention. Metrics should include both retrospective and prospective data and minimize potential confounders. Example metrics can include number of personnel trained, maintenance requests on equipment, procedures completed using equipment, and financial impact. It is vital to carefully choose metrics to be collected, as collection can place undue burden on in-country partners, making its collection less likely. Retrospective data may be sparse, so working with local partners to learn what has been collected previously can aid in prospective metric development. This communication could also reveal efforts that may impact metrics and confound impact assessment. Periodic updates on progress can enable evidence based decision making throughout the process, with continuous engagement of in-country partners. While measuring impact can be more challenging and resource intensive than the donation itself, these efforts are vital to ensure impactful future donations.

## Conclusion

The framework presented for equipment sustainability in developing countries is comprised of four steps: 1) establishing reliable in-country relationships with stakeholders, 2) identifying needs for sustainable solutions locally, 3) exploring potential solutions and assessing their effort-to-impact ratios, and 4) working with strategic partners to implement solutions with clear performance metrics. While intuitive at first, every step involves a nontrivial level of nuance that can significantly hinder progress. Firstly, team members need to be aware that their efforts will likely require a long-term commitment, as bureaucracy and communication chains can slow down implementation time significantly. Projects may take long enough to require careful planning of leadership transitions, ensuring effective communication lines and shifting of responsibilities. Moreover, when selecting which equipment to target first, priority should be given to those that pose the lowest barriers to completion. These types of solutions can shorten the time to impact while enabling teams to become familiar with stakeholders, internal politics, chains of command, and response times. Early displays of success will likely motivate goodwill from stakeholders, potentially impacting downstream projects positively. An example of such a project is discussed further in this issue [3]. Simpler solutions like this may not be immediately obvious, requiring extensive feasibility research. Thus, sufficient time should be allocated to brainstorming and exploring solutions. Self-imposed deadlines may lead to time and resources being invested in subpar solutions requiring more effort and expenses while achieving less impact. It should be expected that many reasonable solutions require skills outside of the expertise of the team. In these scenarios, teams may find it useful to leverage local resources (brand, engineering or media departments, networks) or third-party experts, shifting towards a coordinating role. By focusing on the sustainability of donated equipment instead of the equipment itself, this method presented distinguishes itself from other philanthropic endeavors in the field by seeking to establish preventive maintenance habits that can impact clinical outcomes of a community long-term.
